# MAX-DOAS Measurements of Tropospheric NO_2_ and HCHO Vertical Profiles at the Longfengshan Regional Background Station in Northeastern China

**DOI:** 10.3390/s23063269

**Published:** 2023-03-20

**Authors:** Shuyin Liu, Siyang Cheng, Jianzhong Ma, Xiaobin Xu, Jinguang Lv, Junli Jin, Junrang Guo, Dajiang Yu, Xin Dai

**Affiliations:** 1 State Key Laboratory of Severe Weather & Institute of Tibetan Plateau Meteorology, Chinese Academy of Meteorological Sciences, Beijing 100081, China; 2 State Key Laboratory of Applied Optics, Changchun Institute of Optics, Fine Mechanics and Physics, Chinese Academy of Sciences, Changchun 130033, China; 3 Meteorological Observation Center of China Meteorological Administration, Beijing 100081, China; 4 Longfengshan Regional Background Station, Heilongjiang Meteorological Bureau, Wuchang 150200, China

**Keywords:** NO_2_, HCHO, vertical profile, MAX-DOAS, regional atmospheric background station

## Abstract

The vertical profiles of nitrogen dioxide (NO_2_) and formaldehyde (HCHO) in the troposphere at the Longfengshan (LFS) regional atmospheric background station (127°36′ E, 44°44′ N, 330.5 m above sea level) from 24 October 2020 to 13 October 2021 were retrieved from solar scattering spectra by multi-axis differential optical absorption spectroscopy (MAX-DOAS). We analyzed the temporal variations of NO_2_ and HCHO as well as the sensitivity of ozone (O_3_) production to the concentration ratio of HCHO to NO_2_. The largest NO_2_ volume mixing ratios (VMRs) occur in the near-surface layer for each month, with high values concentrated in the morning and evening. HCHO has an elevated layer around the altitude of 1.4 km consistently. The means ± standard deviations of vertical column densities (VCDs) and near-surface VMRs were 4.69 ± 3.72 ×10^15^ molecule·cm^−2^ and 1.22 ± 1.09 ppb for NO_2_, and they were 1.19 ± 8.35 × 10^16^ molecule·cm^−2^ and 2.41 ± 3.26 ppb for HCHO. The VCDs and near-surface VMRs for NO_2_ were high in the cold months and low in the warm months, while HCHO presented the opposite. The larger near-surface NO_2_ VMRs appeared in the condition associated with lower temperature and higher humidity, but this relationship was not found between HCHO and temperature. We also found the O_3_ production at the Longfengshan station was mainly in the NO_x_-limited regime. This is the first study presenting the vertical distributions of NO_2_ and HCHO in the regional background atmosphere of northeastern China, which are significant to enhancing the understanding of background atmospheric chemistry and regional ozone pollution processes.

## 1. Introduction

Tropospheric nitrogen dioxide (NO_2_) and formaldehyde (HCHO) are two important trace gases [1]. NO_2_ is not only an important air pollutant of photochemical smog and nitric acid rain [1,2], but also one of the precursors of ozone (O_3_), secondary aerosols, and peroxyacetyl nitrate (PAN) [1]. NO_2_ is produced by natural processes (such as lighting and ammonia oxidation [3]) and anthropogenic emission (such as transportation [4], heating/power generation [5] and coal mining [6]). Moreover, nitrogen oxides (NO_x_) production are closely related to the conditions of internal combustion [7]. NO_2_ is chemically removed by photolysis and reactions with various types of free radicals or O_3_ [2]. As the most abundant carbonyl compound in the troposphere, HCHO comes from the combustion of fossil fuels and biomass and the chemical oxidation of volatile organic compounds (VOCs) in the atmosphere [8]. Atmospheric HCHO can be removed by various photochemical reactions [9,10,11]. NO_2_ and HCHO are usually treated as proxies for nitrogen oxides (NO_x_) and VOCs, respectively, to investigate the sensitivity of O_3_ production [12,13].

Tropospheric columns of NO_2_ and HCHO can be measured by satellite remote sensing, such as by the Tropospheric Monitoring Instrument (TROPOMI) [14]. Although TROPOMI has high spatial resolution (5.5 × 3.5 km^2^ at nadir), there are still large uncertainties of retrieved tropospheric NO_2_ and HCHO in the background regions over mountain terrains [15]. For surface NO_2_ and HCHO, there are a few methods to achieve their measurements, such as Fourier transform infrared spectroscopy (FTIR) [16] and long-range active differential optical absorption spectroscopy for NO_2_ [17]. However, the column or surface measurements without the information of NO_2_ and HCHO vertical distributions have limitations in understanding the spatio–temporal variations of NO_2_ and HCHO, which are significant to the investigation of the mechanisms of atmospheric chemistry in regional pollution.

Multi-axis differential optical absorption spectroscopy (MAX-DOAS), a passive ground-based remote sensing observation technique, can be used to measure the vertical column densities (VCDs) and profiles of trace gases in the troposphere [18,19]. It is characterized by high sensitivity, fast response, simple operation, and low operating cost [20]. As early as 2001, MAX-DOAS was used to measure the NO_2_ emissions on highways [21]. Since then, a large number of studies on MAX-DOAS have been conducted in many countries [22]. In recent years, tropospheric vertical profiles of trace gases were successfully achieved by using MAX-DOAS [23]. In the past decade, many studies on NO_2_ and HCHO observed by ground-based MAX-DOAS have been reported in China [24,25,26,27,28,29]. These studies were mainly concentrated in urban and severely polluted areas with large populations and developed economies, such as Beijing–Tianjin–Hebei, Yangtze River Delta, Pearl River Delta, and Sichuan-Chongqing [30,31,32,33,34]. Under the framework of World Meteorological Organization Global Atmosphere Watch (WMO/GAW) in China, MAX-DOAS observations were performed at the background stations of Shangdianzi in the North China Plain and Waliguan in the Tibetan Plateau, revealing the levels and temporal variations of NO_2_ and HCHO in the background atmosphere of the regions [35,36]. However, to our knowledge, no investigation on levels and temporal variations of NO_2_ and HCHO vertical distributions has been made at a background site in the northeastern China, which limits our understanding of the photochemistry and regional pollution formation mechanisms of ozone in this area.

In this study, we performed MAX-DOAS observations at the Longfengshan regional atmospheric background station from October 2020 to October 2021 in order to enhance our understanding of the characteristics of temporal evolutions of NO_2_ and HCHO vertical distribution in the background atmosphere. Section 2 introduces the site and instrument as well as the methods of spectral analysis and profile retrieval of NO_2_ and HCHO. Section 3 presents the temporal variations of NO_2_ and HCHO vertical profiles as well as the sensitivities of O_3_ production to the ratio of HCHO to NO_2_. Finally, the discussion and conclusions are given in Section 4 and Section 5, respectively.

## 2. Experiments and Methods

### 2.1. Site and Instrument

**Figure 1:**
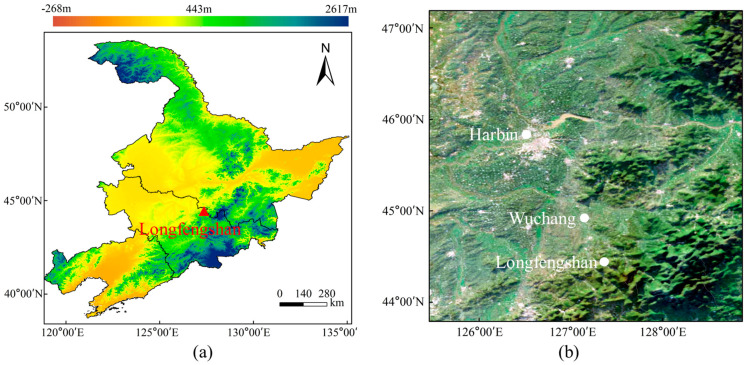
(**a**) The elevation map of Longfengshan regional atmospheric background station. (**b**) The major neighborhood cities around the LFS station (the background image is from the Sentinel−2 satellite).

Ground-based MAX-DOAS measurements were performed at the Longfengshan (LFS) regional atmospheric background station (127°36′ E, 44°44′ N, 330.5 m a.s.l.) from 24 October 2020 to 13 October 2021 (with a break from 10 December 2020 to 3 March 2021 due to instrument malfunction). At present, the LFS station is the only background station under the framework of the WMO/GAW in the Northeast China [37]. The atmospheric observations started in 1991 at LFS [38], which represents the background atmospheric condition in the Northeast Plain region. The LFS station is located on the west side of Longfengshan reservoir, 60 km to the southeast of Wuchang City and 185 km to the southeast of Harbin City (Figure 1). It is located at the intersection of the Songnen plain and the Zhangguangcai ridge of the Changbai mountains. On the western side of the LFS station is the plain area, and on the eastern side is the mountainous forest area [39,40]. There are no villages in the range of 2 km away from the site, no high-density population, and no significant industrial emission sources in the range of 30 km away from the site [41].

We used a mini MAX-DOAS instrument from the Hoffmann Messtechnik GmbH, Germany, which was also used in our previous studies [24,36]. During the Second Cabauw Intercomparison Campaign for Nitrogen Dioxide Measuring Instruments (CINDI-2), the mean relative differences in the slant column from the reference value for the Mini MAX-DOAS were −2.10% for NO_2_ and −20.70% for HCHO [42]. The observation system consisted of an indoor section and an outdoor section. The outdoor section contains the entrance optics, fiber-coupled spectrograph, and controlling electronics inside a metal box. A stepper motor was installed outside the metal box to drive the instrument to rotate in the vertical direction for observation at different elevation angles [19]. In this study, each sequence contained 9 elevation angles (0°, 1°, 2°, 3°, 6°, 10°, 20°, 30°, and 90°), and it took about 4 min for a complete sequence. The spectrograph covered the wavelength range of 290–447 nm. A temperature control module was used to ensure that the spectrograph was operating at stable temperature lower than the ambient temperature. Solar scattering spectra were collected during the daytime period. The spectra of dark current and electron offset were also measured at specific operating temperatures, which were used to correct the measured spectra in the daytime and then reduce the influence of the spectrograph’s photoelectric noise [32]. More information about the instrumentation parameters can be found in the previous studies [31,43,44].

### 2.2. Spectral Analysis

**Figure 2:**
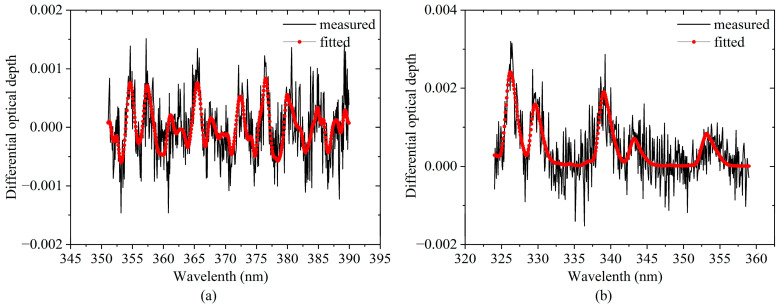
Examples of spectral fitting for (**a**) NO_2_ and (**b**) HCHO. Black and red curves are the measured and fitted differential optical depths, respectively. The NO_2_ and HCHO dSCDs are 1.10 × 10^16^ molecule·cm^−2^ and 2.30 × 10^16^ molecule·cm^−2^, respectively. The RMS between measured and fitted spectra are 7.63 × 10^−4^ for NO_2_ and 8.98 × 10^−4^ for HCHO, respectively.

According to the Beer–Lambert law, the differential slant column density (dSCD) can be retrieved from the measured scattering spectra by the DOAS method [19]. The dSCDs are the differences in slant column densities between the measured and reference spectra. The spectral analysis procedure was implemented by QDOAS software [45]. Before the spectral analysis, the spectra during the observation period were divided into 9 segments based on the operating temperature of the spectrograph. The observed spectra within each segment are corrected by using their corresponding spectra of dark current and electron offset. The high-resolution solar spectral were used for the wavelength calibration. NO_2_ and HCHO dSCDs were retrieved in wavelength ranges of 351~390 nm and 324~359 nm, respectively. The fitting parameters for NO_2_ and HCHO spectral analyses were similar as in previous studies [36,45,46], see Table 1. In this study, in order to improve the credibility of the data, only the spectra with root mean square (RMS) of the spectral fitting residuals < 0.003 and solar zenith angle (SZA) < 75° were retained [46,47]. Under the control of these two thresholds, we retained 70.67% of the NO_2_ profiles and 68.77% of the HCHO profiles. Figure 2 shows an example of spectral fitting for NO_2_ and HCHO dSCDs from the spectrum measured at 10:00 Beijing time (BJ, UTC + 8 h) on 5 July 2021 at an elevation angle of 10°.

### 2.3. Retrieval of NO_2_ and HCHO Vertical Profiles

The vertical profiles of NO_2_ and HCHO VMRs in the lower troposphere (0~4 km) were retrieved from the dSCDs by using the Profile inversion algorithm of aerosol extinction and the trace gas concentration algorithm (PriAM) [47]. The inversion consists of two procedures. In the first step, the vertical profiles of aerosol extinction (AE) were retrieved from the measured O_4_ dSCDs. Then the retrieved AE profiles were used for the inversion of the trace gas profiles [45,47]. For HCHO, the AE profiles at 360 nm were transformed into profiles at 343 nm by using the Ångström exponent. In this study, we set the Ångström exponent as 0.7, which was estimated based on the ERA5 reanalysis data. The entire observation period was divided into six segments according to the working temperature of the spectrograph. The inversion parameters of surface albedo, single scattering albedo, and asymmetry factor were listed in Table 2.

The PriAM algorithm was based on the optimal estimation method to obtain the vertical profiles via loop iteration, which minimized the parameter of cost function [47,52]. The smaller values of the cost function correspond to the more reliable retrieval results. The relative deviations of dSCDs reflect the differences between the PriAM and MAX-DOAS measurements. In order to balance the data quality and quantity, we retained the profiles with cost function < 30 and dSCDs relative deviations < 45% during the post-processing. With respect to the selected thresholds, 61.72% of NO_2_ profiles and 51.71% of HCHO profiles were left.

### 2.4. Ancillary Datasets

The in situ NO_2_ concentration and meteorological data such as surface temperature, relative humidity, and wind speed used in this study were derived from synchronous operational observations at the LFS station. Surface temperature, relative humidity, and wind speed were measured using an automatic meteorological station (DZZ5, Huayun Sonding, China), with precisions of ± 0.1 °C, ± 1%, and ± 0.1 m/s, respectively. The meteorological measurements follow the standard (QX/T 61−2007). The Ångström exponent, surface albedo, planetary boundary layer height (PBLH), and temperature–pressure a priori profiles were obtained from ERA5 reanalysis data (https://www.ecmwf.int/en/forecasts/dataset/ecmwf-reanalysis-v5, accessed on 25 May 2021). The asymmetry factor, single scattering albedo, and Ångström exponent were also referred to the data from the Aerosol Robotic Network (AERONET, https://aeronet.gsfc.nasa.gov/, accessed on 26 May 2021). The instrument used in the AERONET network was the CE318 multi−wavelength solar photometer (CIMEL, Paris, France).

## 3. Results

### 3.1. Overview

**Figure 3:**
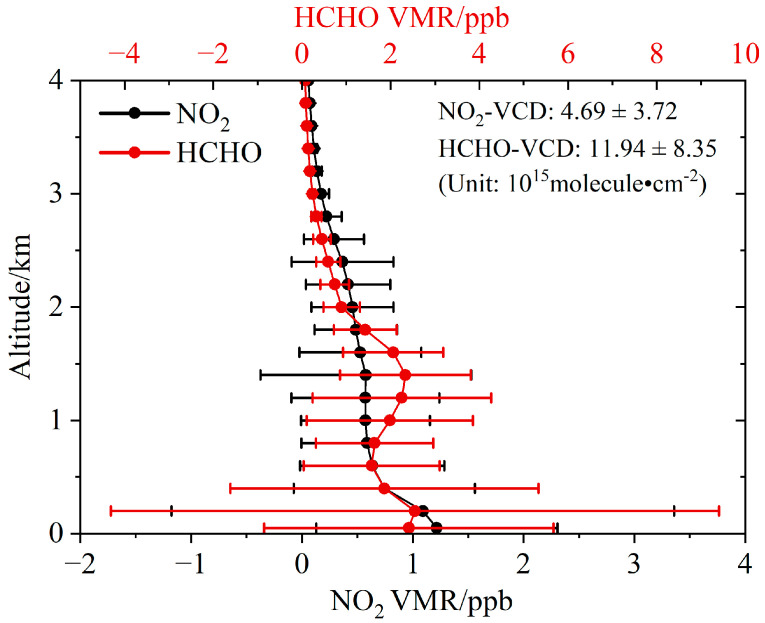
Vertical profiles of NO_2_ (black) and HCHO (red) VMRs. The lines with dots and the error bars indicate the means and standard deviations at different altitudes during the observation period. The averaged values ± standard deviations of tropospheric NO_2_ and HCHO VCDs (Unit: 10^15^ molecule·cm^−2^) during the observation period are also shown in the figure.

The averaged NO_2_ and HCHO vertical profiles below 4 km for the whole observation period are shown by the lines with dots in Figure 3. The NO_2_ VMR has the largest value at the surface and decreases with increasing altitude. There is an elevated HCHO pollution layer with VMRs larger than 2 ppb around the altitudes of 1.4 km. The means ± standard deviations of NO_2_ and HCHO VCDs are 4.69 ± 3.72 × 10^15^ and 1.19 ± 0.84 × 10^16^ molecule·cm^−2^, respectively. Correspondingly, the near-surface VMRs are 1.22 ± 1.09 ppb for NO_2_ and 2.41 ± 3.26 ppb for HCHO. The near-surface VMRs of NO_2_ and HCHO in this study are higher than those of NO_2_ (7~100 ppt) and HCHO (0.9 ppb) at the Waliguan regional atmospheric background station from 2012 to 2015 [36].

### 3.2. Temporal Variations

#### 3.2.1. Monthly Variations

**Figure 4:**
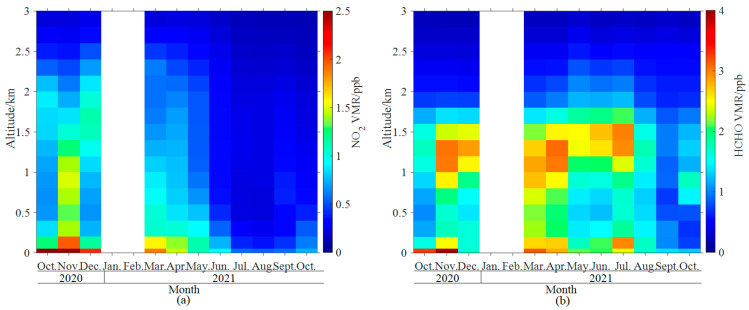
Monthly vertical profiles of (**a**) NO_2_ and (**b**) HCHO VMRs.

The monthly variations of the vertical profiles were different between NO_2_ and HCHO (Figure 4). The larger NO_2_ VMRs occur at the near-surface and decrease with increasing altitudes each month. The NO_2_ VMRs were lower in the lower troposphere in summer than in other seasons. For HCHO, larger VMRs appear not only near the surface but also at upper levels (~1.4 km altitude) in most observational months.

**Figure 5:**
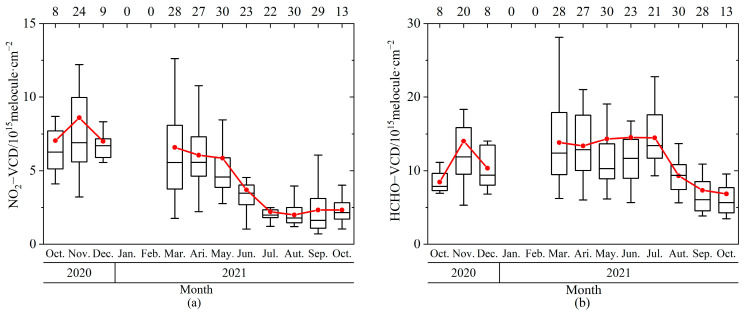
Monthly tropospheric VCDs (Unit: 10^15^ molecule·cm^−2^) for (**a**) NO_2_ and (**b**) HCHO. The upper (lower) error bars and upper (lower) boundaries of the boxes are the 95th (5th) and 75th (25th) percentiles of the data grouped per month, respectively. The lines inside the boxes and the red curves with dots indicate the medians and the averages, respectively. The number of sampling days per month is marked on the top axis.

The maxima (minimum) of monthly averaged NO_2_ near-surface VMRs (Figure 4a) and VCDs (Figure 5a) were 2.51 ppb (0.47 ppb) and 8.6 × 10^15^ molecule·cm^−2^ (2.00 × 10^15^ molecule·cm^−2^) in November 2020 (August 2021). Tropospheric NO_2_ VCD shows a monthly variation trend of being higher in colder months and lower in warmer months (Figure 5a). The open burning of straw in autumn and the burning of fuel for heating in winter probably lead to an increase in NO_2_ emissions compared to summer. The cold and dry atmospheric conditions in autumn and winter are not conducive to gas spreading, resulting in the highest NO_2_ concentrations in autumn and winter [53].

The monthly HCHO VCDs maintained a high level from March to July 2021, with a maximum of 1.45 × 10^16^ molecule·cm^−2^ in June (Figure 5b). The HCHO minimum for both near-surface VMRs (1.12 ppb, Figure 4b) and VCDs (6.83 × 10^15^ molecule·cm^−2^, Figure 5b) appeared in October 2021. The near-surface HCHO VMRs were enhanced from late October to early December 2020, probably due to increased primary HCHO emissions from fossil fuel combustion in North China during the heating period [54]. The HCHO vertical distribution and column were influenced by complex factors, such as primary source from fossil fuel combustion [8] and secondary sources from chemical oxidation reactions [9]. The causes of HCHO temporal variation should be further investigated by comprehensive observation in future.

#### 3.2.2. Diurnal Variations

**Figure 6:**
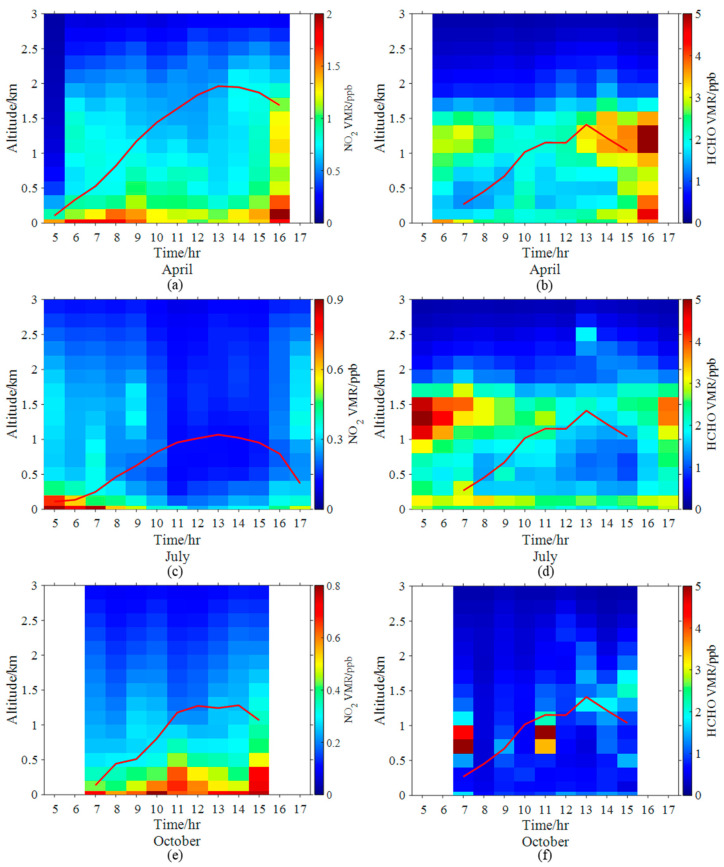
Diurnal variations of the vertical profile of NO_2_ in April (**a**), July (**c**) and October (**e**). (**b**,**d**,**f**) Same as (**a**,**c**,**e**) correspondingly, but for HCHO. The red line indicates the planetary boundary layer height (PBLH).

In this study, we use April, July, and October 2021 as representatives of spring, summer, and autumn, respectively. The sample number in winter was too small to be discussed here. Figure 6 shows the diurnal variations of the vertical profile of NO_2_ and HCHO VMRs in April, July, and October 2021. High near-surface NO_2_ VMRs occurred in the early morning (5:00–9:00 BJ) and late evening (15:00–17:00 BJ) during April and July, but were stable throughout the daytime in October. In all three months, a polluted layer of NO_2_ was present in the residual layer. The planetary boundary layer developed to a maximum at 13:00 BJ. The elevated layer of NO_2_ disappeared gradually as the planetary boundary layer developed. The elevated layer existed throughout the day. Active photochemical reactions in the upper planetary boundary layer can contribute to the persistence of the HCHO elevation layer in the afternoon [55]. This characteristic is consistent with our observations at Raoyang meteorological station [46].

**Figure 7:**
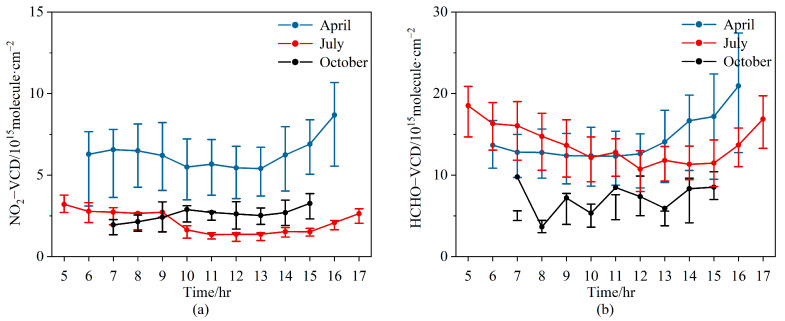
Diurnal variations of tropospheric VCDs for (**a**) NO_2_ and (**b**) HCHO. The dots denote the averages, and the error bars represent the 25th and 75th percentiles of the data grouped for each hour. The blue, red, and black dotted lines denote April, July and October 2021, respectively.

The daily variations of NO_2_ and HCHO VCDs are shown in Figure 7. NO_2_ VCDs remained at almost the same level throughout the day in April, with a slight increase after 14:00 BJ. In July, NO_2_ VCDs slightly decreased from 5:00 to 9:00 BJ, stayed almost the same from 10:00 to 15:00 BJ, and then increased slightly after 16:00 BJ. In October, NO_2_ VCDs were nearly constant throughout the day. The NO_2_ diurnal patterns were different from those in urban areas, where there are two peaks in the morning and evening due to traffic emissions [56]. For all three months, the NO_2_ diurnal variations ranges were small, implying that the local anthropogenic emission and photochemical reactions were weak at the LFS background station.

**Figure 8:**
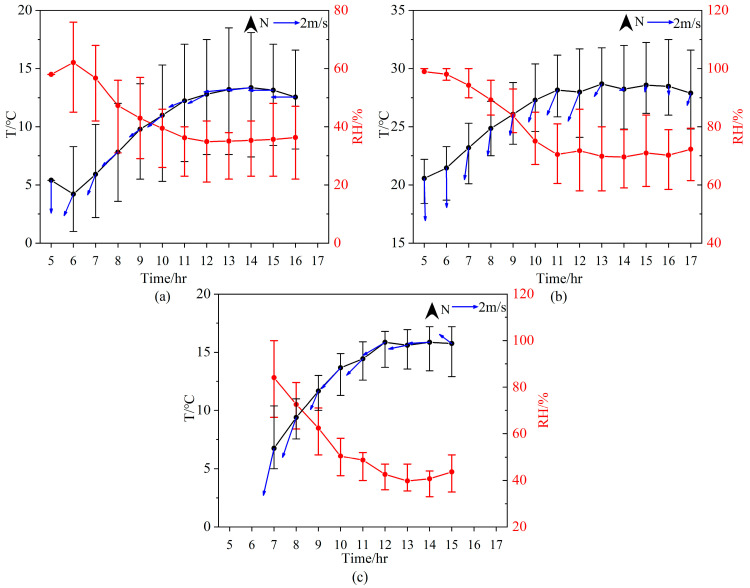
Diurnal variations of temperature (black), relative humidity (red), wind speed and direction (blue) in (**a**) April, (**b**) July and (**c**) October 2021.

In April, HCHO VCDs varied gently during the period from 06:00 to 12:00 BJ and started to increase at 12:00 BJ until they reached the highest value at 16:00 BJ. In July, HCHO concentrations were high in the morning and evening but low at noon, with the maximum value appearing at 05:00 BJ. HCHO VCDs decreased to a minimum at 12:00 BJ and remained stable until 15:00 BJ. By comparing with the corresponding temperature and relative humidity qualitatively (Figure 8), we found that high NO_2_ VCDs tended to occur at low temperatures and high humidity, but this relationship was not found between HCHO and temperature. Because of the low wind speeds, the LFS station was mainly influenced by emissions from local sources.

### 3.3. HCHO/NO_2_ Ratio

#### 3.3.1. Temporal Variation

The production of tropospheric ozone (O_3_) in most regions is associated with VOCs and NO_x_ [57]. The ratio of HCHO to NO_2_ is an effective indicator for investigating the sensitivity of chemical ozone production to nitrogen oxides (NO_x_) and VOCs [46,58,59]. “RA” is used to refer to the ratio of HCHO to NO_2_, either in VMRs (RA_VMR_) or tropospheric VCD (RA_VCD_). According to previous studies, O_3_ production can be divided into VOCs-limited (RA < 1), transition zone (1 < RA < 2) and NO_x_-limited (RA > 2) [60,61]. In this study, in order to balance the quality and quantity of the data, only HCHO VMRs between 1 and 10 ppb and NO_2_ larger than 0.1 ppb were considered for the calculation of RA [46].

**Figure 9:**
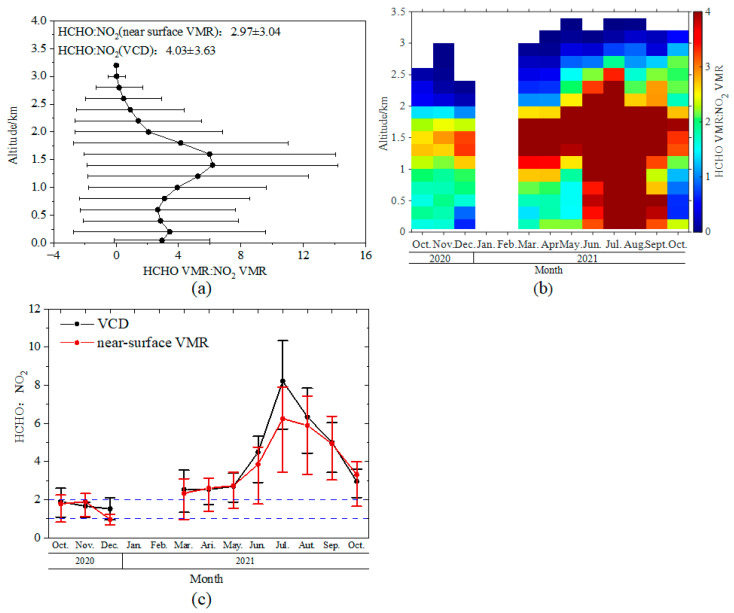
(**a**) Vertical profiles of the ratio of HCHO to NO_2_ during the observation period. The dots and whiskers indicate the average of all data at selected altitudes during the observation period and the standard deviation, respectively. (**b**) Monthly variation of vertical profiles of VMRs ratios of HCHO to NO_2_. (**c**) Monthly variation of the ratio of HCHO to NO_2_ for tropospheric VCDs and near-surface VMRs. The dots and the error bars denote the averages and the 25th and 75th percentiles of the data grouped for each month. HCHO/NO_2_ = 1 and 2 are also marked with dashed lines. The white grid cells in panel (**b**) indicate the missing data owing to lacking observation and data quality control.

RA with respect to VMR has an elevated layer at the altitude of around 1.5 km (Figure 9a). The vertical distribution of the monthly means of RA (Figure 9b) shows that the height of the lower boundary of the elevated RA fell from October 2020 (1.2 km) to May 2021 (0.8 km). The larger RA filled the entire lower troposphere (below 2 km) from June until September 2021. In October 2021, RA in the lower troposphere decreased, and the lower boundary of the elevated layer rose again to 1.2 km. The majority of RA were less than 2 above the altitude of 2 km and less than 1 above the altitude of 2.5 km. The RAs of near-surface VMR and tropospheric VCD had the same seasonal variation patterns, with annual averages of 2.97 ± 3.04 for near-surface VMR and 4.03 ± 3.63 for tropospheric VCD (Figure 9c). The largest values of both RA_VMR_ and RA_VCD_ appeared in July, being 6.25 ± 4.00 and 8.22 ± 3.62, respectively. Correspondingly, the lowest RA appeared in December with the values of 0.96 ± 0.36 for RA_VMR_ and 1.53 ± 0.67 for RA_VCD_.

**Figure 10:**
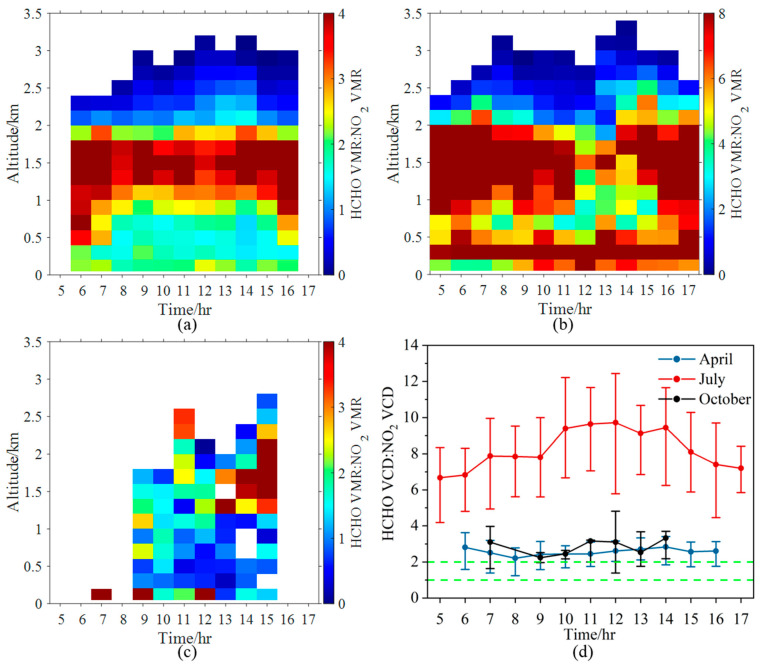
Diurnal variations in the vertical profile of the ratio of HCHO to NO_2_ VMRs in (**a**) April, (**b**) July, and (**c**) October 2021. (**d**) Diurnal variations of RA_VCD_ in April, July, and October 2021. The dots and error bars represent the averages and the 25th (75th) percentiles of the data grouped for each hour in April (blue), July (red) and October (black).

The diurnal variation patterns of RA vertical distribution varied with seasons (Figure 10). In each month, the elevated layer of RA appeared around the altitudes of 1.5 km. In April, the RA was mainly between 1 and 2 in the lower boundary layer (0–0.4 km), with RA < 1 above 2.5 km. In July, RA > 2 filled the entire boundary layer (up to 2 km), with RA < 2 only appearing above 2 km. In October, RA was almost between one and two near the surface. The RA_VCD_ was larger than two in all the three months. On average, the RA in July was ~3.2 and ~2.8 times higher than that in April and October, respectively.

#### 3.3.2. Implication for O_3_ Production

**Figure 11:**
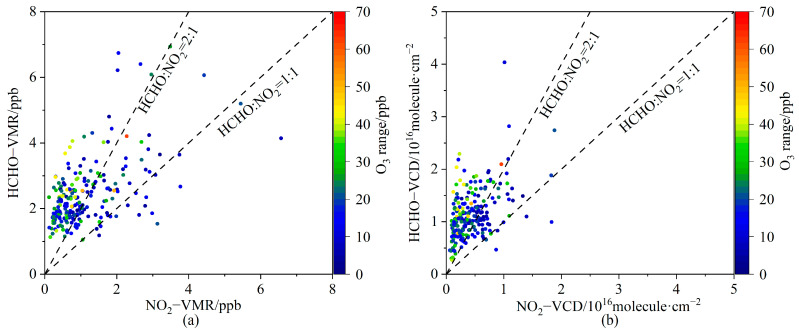
(**a**) Daily average of near-surface VMRs for HCHO and NO_2_. (**b**) same as (**a**) but for VCDs, colored by the range of daytime surface O_3_ variation. The dashed lines indicate HCHO/NO_2_ = 1 and 2.

To analyze the sensitivities of O_3_ production to the ratio of HCHO to NO_2_, we assume that daily O_3_ production at the LFS is approximately equal to the range of daytime O_3_ concentration variations at the surface, considering that the photochemical O_3_ production was a strong driver of the diurnal surface O_3_ variation [46,59]. Figure 11 shows the daily O_3_ production as a function of the daily average values of HCHO and NO_2_ for near-surface VMR (Figure 9a) and VCD (Figure 9b) during the daytime (05:00 to 17:00 BJ). The distribution patterns of O_3_ production, as classified by RA_VMR_ and RA_VCD_, are similar. For most of the days, O_3_ production was concentrated in the NO_x_-limited regions or transition regions. On only a few days was the weaker O_3_ production (<20 ppb d^−1^) located in the VOCs-limited regions. Differently, the strongest near-surface O_3_ production occurred in the transition region for RA_VMR_, but in the NO_x_-limited region for RA_VCD_. From October 2020 to March 2021, the RA_VMR_ varied between 1 and 2, implying that O_3_ production was in the transition regime. As a whole, O_3_ production was in the NO_x_-limited regime.

#### 3.3.3. Relationship to Meteorological Conditions

**Figure 12:**
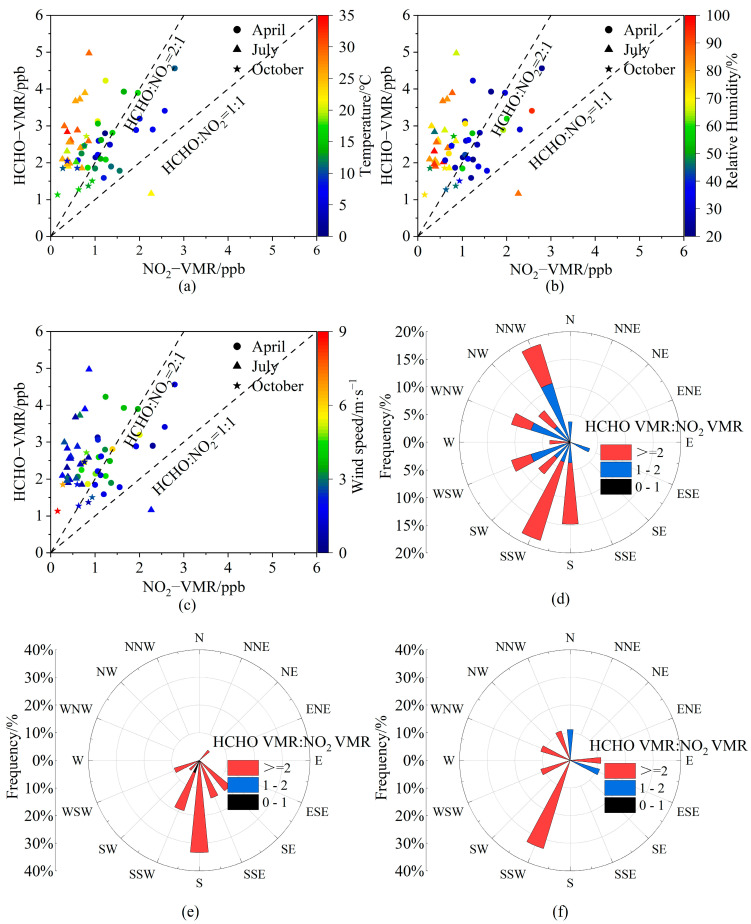
Relationship between the near-surface VMR ratios of HCHO to NO_2_ and meteorological factors, i.e., (**a**) air temperature, (**b**) relative humidity, (**c**) wind speed, and (**d**−**f**) wind direction. The dots, triangles, and pentagrams represent April, July, and October 2021, respectively. Rose plots denote RA_VMR_ frequencies in 16 wind directions in (**d**) April, (**e**) July and (**f**) October 2021.

The correlation coefficient between RA_VMR_ and temperature (relative humidity) is 0.62 (0.53), implying that O_3_ production at LFS tended to be in the NOx−limited region when the temperature and relative humidity were higher (Figure 12a, b). In July, the temperature and relative humidity were high, and the RA was basically larger than two. There is no correlation relationship between RA_VMR_ and wind speed (Figure 12c), with the correlation coefficient of 0.01 (not shown in figure). However, the RA_VMR_ is dependent on the wind directions (Figure 12d–f). In April, maximum frequencies for RA_VMR_ ≥ 2 and 1 < RA_VMR_ < 2 were 14.81% in the SSW sector and 11.11% in the NNW sectors. Almost all the RA_VMR_ values in July were larger than two and distributed in the SE–WSW sectors, with the largest frequency occurring in the S direction. In October, the largest frequency for RA_VMR_ > 2 was 33.33% in the SSW direction and the frequencies for 1 < RA_VMR_ < 2 were smaller and in the N and ESE directions. As a whole, the sensitivity of O_3_ production to NO_2_ and HCHO is closely related to temperature and relative humidity. Air masses from different directions can lead to different sensitivities of O_3_ production to NO_x_ and VOCs at LFS Station.

## 4. Discussion

The averaged NO_2_ VCDs (4.69 × 10^15^ molecule·cm^−2^) during our observation period are larger than the mean level (1.16 × 10^15^ molecule·cm^−2^) in Heilongjiang province from 2005 to 2015 [60]. However, the mean HCHO VCDs in this study (1.19 × 10^16^ molecule·cm^−2^) are lower than those (1.39 × 10^16^ molecule·cm^−2^) in Heilongjiang province from 2005 to 2018 [61]. The background levels of NO_2_ and HCHO at LFS are useful to investigate as long-term trends in future studies.

The ranges of diurnal variation of NO_2_ VCDs are smaller at the LFS station than at another WMO/GAW background region in the North China Plain [35]. The vertical profile structures of both NO_2_ and HCHO at LFS in summer are similar to those observed at Raoyang, a rural site in the North China Plain [46]. The MAX−DOAS measurements at Raoyang were performed only for a summer period, and the elevated HCHO layer can be attributed to the air pollution and oxidization process in the lower atmosphere in the North China Plain [55]. The causes of larger VMRs at upper levels (~1.4 km altitude) at the LFS background station need to be fully understood in future studies.

Meteorological conditions affected the concentration levels, vertical distribution, and temporal variations of NO_2_ and HCHO, as well as the sensitivity of O_3_ production at the LFS station. There are at least three reasons: (1) The boundary layer evolution led to the vertical mixing of air masses; (2) The temperature and relative humidity affected the efficiency of O_3_ photochemical processes and the natural source emission; (3) Different wind speeds and directions were accompanied by air masses with different pollution levels.

## 5. Conclusions

We carried out MAX−DOAS observations at the Longfengshan regional atmospheric background station in Heilongjiang Province of China from October 2020 to October 2021, performed the spectral analysis with QDOAS software, and retrieved vertical profiles of NO_2_ and HCHO by using the PriAM algorithm. We explored the concentration, temporal, and spatial variation of NO_2_ and HCHO, and the sensitivity of O_3_ production to the ratio of HCHO to NO_2_. The conclusions are as follows:

The average levels of near-surface VMRs for NO_2_ and HCHO were 1.22 ± 1.09 ppb and 2.41 ± 3.26 ppb throughout the observation period, respectively. The NO_2_ vertical structure shows a declining pattern with increasing altitude, while HCHO has an elevated layer around the altitude of 1.4 km.

The NO_2_ VMRs in the lower troposphere tend to be higher in colder months and lower in warmer months at the LFS station. HCHO has an opposite monthly trend, especially at upper levels (~1.4 km altitude). There was a polluted layer of NO_2_ in the residual layer, which disappeared gradually with the development of planetary boundary layer. However, the HCHO elevated layer maintained throughout the daytime. Different from the gentle diurnal variations of NO_2_ VCDs, the HCHO VCDs are high in the morning and evening but low at noon in July. The qualitative comparisons show that high NO_2_ VCDs tended to occur at low temperatures and high humidity, but this relationship is not found for HCHO.

Although there were monthly and diurnal variations in the vertical profiles of the VMR ratio of HCHO to NO_2_, on most of the days, O_3_ production was in the NO_x_-limited regions or transition regions at the LFS station. There were only a few days of the experiment time when the weaker O_3_ production (<20 ppb) was located in the VOCs-limited regions. Besides the dependencies of wind direction, the O_3_ production at the LFS tended to be in the NOx-limited region when the temperature and relative humidity were higher.

The results of this study will be helpful for better understanding the spatiotemporal distribution characteristics of NO_2_ and HCHO in the background region, in addition, more comprehensive observations, such as concurrent measurements of O_3_ and VOCs together with MAX-DOAS, are recommended, which are significant for evaluating and designing the strategies for controlling O_3_ pollution.

## Figures and Tables

**Table 1:** Setting for NO_2_ and HCHO spectral analyses.

Parameters	O_4_ and NO_2_	HCHO
Fraunhofer reference	sequential spectra	
Fitting interval	351~390 nm	324~359 nm
DOAS polynomial	degree: 5	
Intensity offset	degree: 2 (constant and order 1)	
Shift and stretch	spectral	
Ring spectra	Original and wavelength-dependent Ring spectra	
NO_2_ cross section	Vandaele et al. (1998) [48], 294 K, 220 K, I_0_ correction (10^17^ molecule·cm^−2^)	Vandaele et al. (1998) [48], 294 K, I_0_ correction (10^17^ molecule·cm^−2^)
O_3_ cross section	Serdyuchenko et al. (2014) [49], 223 K, I_0_ correction (10^20^ molecule·cm^−2^)	Serdyuchenko et al. (2014) [49], 223 K, 243 K, I_0_ correction (10^20^ molecule·cm^−2^)
O_4_ cross section	Thalman and Volkamer (2013) [50], 293 K	
HCHO cross section	Meller and moortgat (2000) [51], 298 K	

**Table 2:** Surface albedo, single scattering albedo, and asymmetry factor for each segment of profile inversion.

NO.	Start and End Time	Surface Albedo	Single Scattering Albedo	Asymmetry Factor
1	2020/10/26—2020/11/18	0.11	0.94	0.7
2	2020/11/19—2020/12/08	0.22	0.94	0.7
3	2021/03/04—2021/04/07	0.13	0.93	0.7
4	2021/04/07—2021/06/12	0.15	0.92	0.7
5	2021/06/13—2021/07/27	0.17	0.94	0.7
6	2021/08/01—2021/10/12	0.15	0.96	0.7

## Data Availability

Data sharing not applicable.

## Supplementary Materials

The following supporting information can be downloaded at: https://www.mdpi.com/1424-8220/23/6/3269/s1.
