# Supplementary material for: MAX-DOAS Measurements of Tropospheric NO2 and HCHO Vertical Profiles at the Longfengshan Regional Background Station in Northeastern China
Source: Sensors (Basel). 2023 Mar 20;23(6):3269. doi: 10.3390/s23063269 (PMC10099724; doi:10.3390/s23063269)
Supplement: Supplementary file 1 [file sensors-23-03269-s001.zip › sensors-2227796-supplementary.pdf]

sensors

MDPI

Article

Supplementary Materials: MAX-DOAS Measurements of Tropospheric NO2 and HCHO Vertical Profiles at the Longfengshan Regional Background Station in Northeastern China

Shuyin Liu [1], Siyang Cheng [1,2,*], Jianzhong Ma [1], Xiaobin Xu [1], Jinguang Lv [2], Junli Jin [3], Junrang Guo [1], Dajiang Yu [4] and Xin Dai [4]

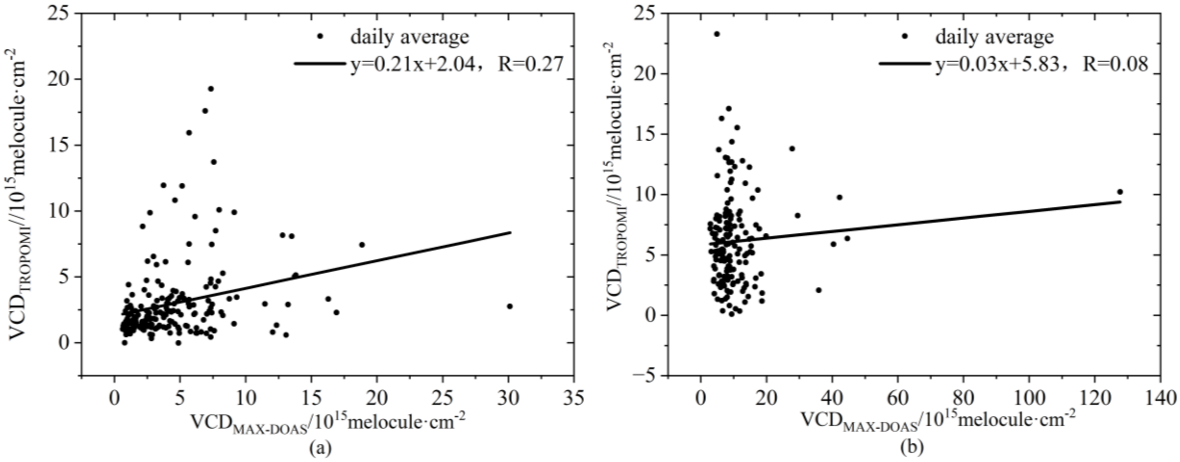

Figure S1. (a) Correlation between the daily averages of NO_2 VCDs retrieved by TROPOMI satellite and MAX-DOAS. **(b)** Same as **(a)**, but for HCHO. The lines indicate the linear fit between the two data sets.

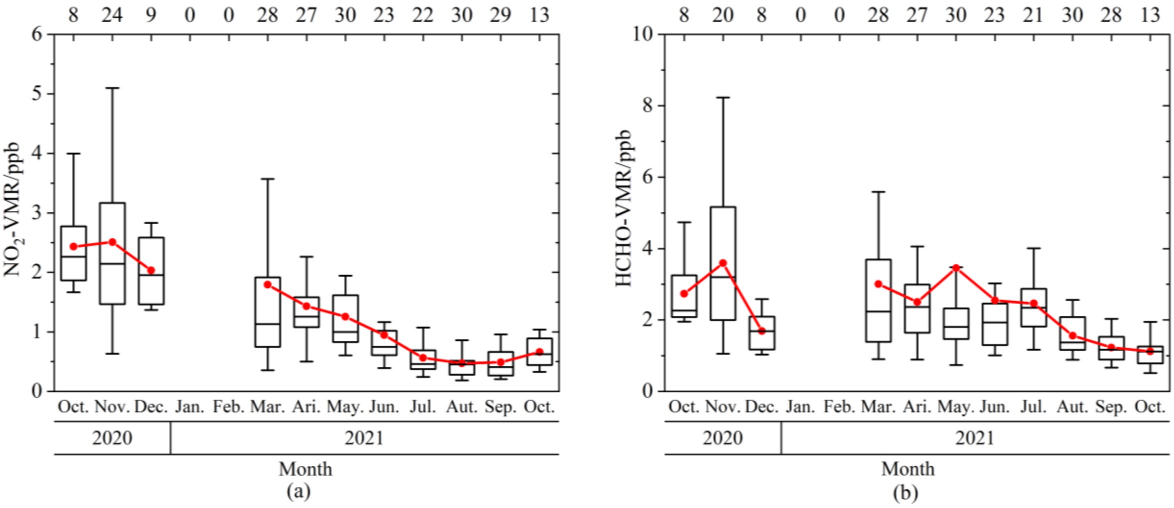

Figure S2. Monthly near-surface VMRs for **(a)** NO_2 and **(b)** HCHO. (Unit: ppb). The upper (lower) error bars and upper (lower) boundaries of the boxes are the 95th (5th), 75th (25th) percentiles of the data grouped per month, respectively. The lines inside the boxes and the red curves with dots indicate the medians and the averages, respectively. The number of sampling days per month is marked on the top axis.

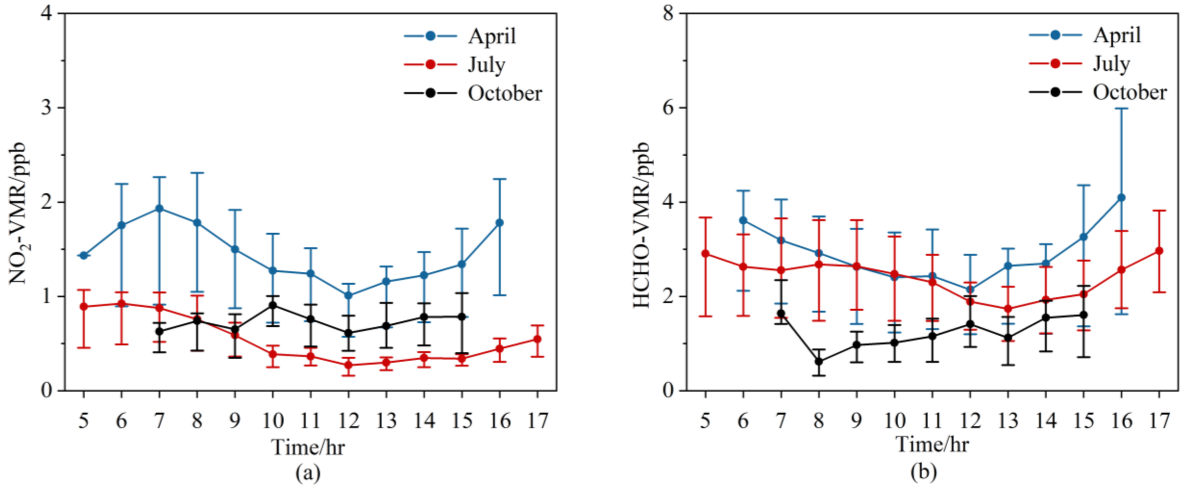

Figure S3. Diurnal variations of near-surface VMRs for **(a)** NO_2 and **(b)** HCHO. The blue, red and black lines with dots denote the averages in April, July and October 2021, respectively. The error bars represent the 25th and 75th percentiles of the data grouped for each hour.
